# Integrated behavioral health services in pediatric primary care and emergency department utilization for suicide risk

**DOI:** 10.3389/fpsyt.2023.1241642

**Published:** 2023-11-09

**Authors:** Brianna C. M. Wellen, Naomi M. Wright, Mira A. Bickford, Eliza Hayes Bakken, Andrew R. Riley

**Affiliations:** ^1^Department of Pediatrics, Oregon Health & Science University, Portland, OR, United States; ^2^Department of Psychiatry and Behavioral Sciences, University of Minnesota, Minneapolis, MN, United States; ^3^Build EXITO Scholars Program, Portland State University, Portland, OR, United States

**Keywords:** integrated behavioral health, adolescent suicide, primary care, screening, pediatrics

## Abstract

**Introduction:**

Universal screening for suicide risk in primary care settings is a promising avenue for preventing self-harm and improving health outcomes. Triaging youth to an appropriate level of care, including diverting lower-risk patients from the emergency department (ED) is a meaningful goal. Previous research indicates integrated behavioral health (IBH) may prevent unnecessary admission to the ED on the day of suicide risk screening. We hypothesized that youth who received an IBH consultation the same day as suicide risk screening would be less likely to be admitted to the ED, but more likely to contact IBH services and utilize primary care in the following month.

**Methods:**

We conducted a retrospective chart review of 3,649 youth aged 10-18 years who were screened with the Ask Suicide-Screening Questions (ASQ) in two pediatric primary care practices. We collected demographic data, ASQ and Patient Health Questionnaire-9 (PHQ-9) scores, as well as patient contacts with IBH, the ED, and medical primary care the day of screening and the following 31 days. We conducted a series of logistic regressions and chi-square analyses to determine whether contact with IBH on the same day as positive suicide risk screenings predicted same-day admission to the ED, IBH contact, and medical primary care utilization.

**Results:**

Among the 7,982 ASQ scores, 1,380 (18%) were non-acute and 87 ASQs (1%) screened acutely positive. Over 90% of positive screens were diverted from the ED regardless of IBH contact. None of the patients died from suicide. Same-day IBH was associated with higher likelihood of general ED visits for all positive screens (acute and non-acute together). None of the positive screens that received an IBH consultation on the same day as screening were admitted to the ED in the subsequent month. Contact with IBH the same day as screening positively predicted utilization of IBH and medical primary care services in the subsequent month, especially for youth with minority race and ethnicity identities.

**Discussion:**

In the context of clinics with IBH and systematic risk assessment processes, most youth who screen positive for suicide risk are diverted from the ED. However, contrary to our hypothesis, our study showed that youth who received same-day IBH consultations were more likely to be admitted to the ED compared to peers who did not receive IBH consultations. These findings suggest that systematic suicide screening combined with IBH consultations in pediatric primary care can effectively identify risk levels and triage patients to appropriate care.

## Introduction

Youth suicide rates in the United States (U.S.) have risen over the past decade; in 2021, 22% of high school students reported seriously considering attempting suicide, 18% made a plan, and 10% made at least one suicide attempt (1). Universal screening for suicide risk in primary care settings is a promising avenue for identifying at-risk youth and improving health outcomes (2, 3); however, screenings identify varying levels of risk that necessitate a range of clinical responses. Evidence-based based guidelines for assessing and responding to suicide risk in pediatric settings exist (4), but primary care providers (PCPs) report significant barriers, including time pressures, lack of training, and limited resources for managing positive screens (5, 6). When patients screen positive in primary care settings and providers do not have sufficient time or expertise to fully assess risk, youth with a wide range of risk may be referred to an emergency department (ED) out of an abundance of caution (7).

Although appropriate for those with imminent risk, ED environments may not be wellsuited for assessing and supporting the mental health needs of most patients with suicidality due to a focus on managing acute medical conditions and a lack of mental health professionals onsite. Youth and their families report experiencing long wait times and insufficient care (8–10) and may leave before being evaluated (11). Beyond suboptimal patient experience, responding to BH concerns in the ED setting is costly and inefficient. The average duration of BH encounters in the ED is significantly longer than other ED visits [nearly twice as likely to be over 4 h (12)]. One study estimated the cost of caring for one BH patient to be $219 per hour, with little apparent value to the patient (8). Further, many youth who present to the ED with behavioral health (BH) concerns do not receive a referral to follow-up services (13).

Given the apparent mismatch between the goals and resources available in the ED and the needs of youth with less than imminent suicide risk, accurately diverting lower-risk patients from the ED, while assuring an appropriate level of care, is a meaningful goal. In the case of primary care-based suicide risk screening, integrated behavioral health (IBH) services that incorporate mental health professionals into the risk assessment process have been proposed as a method to accomplish this goal (7, 14). One promising study found that 93% of patients who screened positive for suicide risk were diverted from the ED following a consultation with a BH specialist, resulting in a considerable estimated cost-savings (7); however, that study was limited by lack of a comparison group and use of a depression screener with suboptimal sensitivity for detecting suicide risk (15). Further, that study focused only on ED visits that occurred the same day as suicide risk screening, so subsequent utilization of the ED and other healthcare, including follow-up mental health supports, was unknown. Understanding the impact of IBH on healthcare besides ED visits is important, because while all patients who screen positive for suicide risk do not require emergency care, they may benefit from ongoing monitoring or intervention. Continuity of care is a core function of primary care (16), as well as an important aspect of suicide prevention (17), so ongoing contact with at-risk patients is desirable.

To address this gap in the literature, we conducted a retrospective analysis of youth patients who screened positive for suicide risk in pediatric primary care, comparing those who received a same-day IBH consultation with those who did not. In addition to same-day ED visits, we assessed outcomes from the subsequent 31 days, including ED visits, IBH contacts, and primary care utilization. We hypothesized that youth who received an IBH consultation the same day as screening would be less likely to be admitted to the ED, but more likely to contact IBH services and to utilize primary care in the following 31 days.

## Method

### Participants

Participants were patients ages 10–18 years who were screened for suicide risk in two academic medical center-affiliated pediatric primary care practices in the Portland, Oregon metropolitan area. One clinic was hospital-based, and the other was community-based. Screening data from December 2019—July 2022 were collected via a retrospective chart review and extracted from the electronic health record using Epic Data Warehouse. Before this period, suicide screening results were not entered into the electronic health record in a manner that could be reliably extracted.

### Setting and integration model

Both clinics were staffed by attending and resident physicians. IBH staffing consisted of licensed clinical social workers, clinical social work associates, psychologist postdoctoral fellows, psychology interns, and psychiatry consultants. Exact levels of IBH staffing and availability fluctuated, and in-person consultation was limited during portions of 2020 and 2021 due to COVID-19 precautions. The integration model at both clinics approximated Level 5 of the Substance Abuse and Mental Health Services Administration-Health Resources and Services Administration Standard Framework for Levels of Integrated Healthcare (18), with clinical and care management services provided both concurrent with medical care, as well as separate from medical care in a “co-located” fashion. Telehealth IBH services were available starting in April 2020, with IBH staff available for both “on demand” and scheduled consultations with patients.

Global screening for suicide risk at both clinics using the Ask about Suicide Questions (ASQ) tool was first implemented in February 2019, with a goal of screening every patient aged 10 years or older at every medical visit. The approach to screening and secondary assessment was consistent with the Zero Suicide Model (19) and informed by established recommendations for managing suicide risk in pediatric settings (4, 20). The framework for assessing and responding to risk was meant to provide a flexible model with a range of outcomes that could be followed by both medical and IBH providers, ideally in a team-based fashion. Social workers were considered first-call for consulting on suicide risk with other members of the IBH team available to assist as needed. Physicians were encouraged to facilitate IBH involvement at any level of risk. Table 1 displays examples of risk indicators and potential clinical responses.

**Table 1:** Clinical framework for evaluating and responding to suicidal risk.

**Continuum of risk**	**Example indicators**	**Possible range of responses**
Low	Passive or historical ideation	Engage IBH team via immediate consultation or referral
	No expressed plan or intent	Provide resources/referrals
	No history or self-harm	Discuss importance of communicating with caregivers
	Significant protective factors	Encourage connection with any existing mental health supports
Low to moderate	Passive ideation concurrent with moderate emotional distress	Engage IBH team via immediate consultation or referral
	History of active ideation but no attempt or self-harm	Consider breaking confidentiality to inform caregivers, ideally with youth assent
	Social withdrawal	Safety plan and lethal means counseling
	Moderate protective factors	Verify follow-up with existing mental health provider within 7 days Schedule follow-up with medical or IBH team within 7 days
Moderate to high	Very recurrent or current ideation	Engage IBH team via immediate consultation
	Specific plan	Break confidentiality to inform caregivers regardless of youth assent
	Access to lethal means	Safety plan and lethal means counseling
	History of self-injury or aborted attempts Stated intent	Consider need for psychiatric admission or crisis stabilization
	Substance abuse	
	Low protective factors	Direct to emergency department for further management

IBH, integrated behavioral health.

### Measures

#### Demographics

Demographic data included age, race, ethnicity, biological sex assigned at birth, and type of insurance. We also attempted to collect gender identity for all participants, but it was available in < 10% of cases. Insurance was categorized as either commercial or public and was the most relevant proxy available for socioeconomic status.

#### Suicide risk screening

The Ask Suicide-Screening Questions (ASQ) tool is a brief suicide risk screening instrument designed for use in medical settings with strong sensitivity and specificity for youth aged 10–21 years (21). The ASQ consists of 4 initial yes/no items. Any score ≥ 1 is considered a positive screen. In the event of a positive screen, a fifth item (“Are you having thoughts of killing yourself right now?”) assesses acute ideation (acute positive screen). The ASQ has been widely used in previous studies and is a reliable and valid measure of suicide risk in pediatric settings (22).

#### Depressed mood

The Patient Health Questionnaire-9 (PHQ-9) is a commonly used self-report screening instrument for symptoms of depression (23). The PHQ-9 consists of nine items that correspond to the nine diagnostic criteria for major depressive disorder, and the language has been slightly adapted for use in pediatric settings. The PHQ-9 and its adaptation for adolescents possess strong psychometric properties and has demonstrated utility in pediatric settings (23–26). The adolescent version (25) was used in the current study, and was intended to be administered at the same visits as ASQ. We were unable to analyze responses to individual PHQ-9 items, as only total scores were archived in the medical record.

#### Healthcare utilization

We recorded all ED visits, IBH encounters, and primary care physician (PCP) encounters that occurred within 31 days of an ASQ screening. Encounters that occurred the same date as ASQ screening were deemed “same-day,” and subsequent utilizations were deemed “31-day.” For ED visits, we also categorized the reason for each admission into either non-suicide related or general mental health challenges (e.g., panic attack), or suicide-related concerns. Two of the authors independently reviewed the admission documentation in the medical record and determined the reason for admission. Interrater agreement after initial coding was strong (Cohen's ℵ = 0.93). Discrepancies were discussed and resolved. Given the ultimate number of recorded visits, we dichotomized reason for admission into “suicide-related” or “other.”

#### Mortality

We queried the data warehouse for any instances of mortality in the sample. We additionally cross-referenced each patient with state-level surveillance data from the Oregon Health Authority to identify any deaths that occurred outside of the medical system.

#### Analytic plan

We used descriptive statistics to characterize the sample. We determined a set of binomial logistic regression analyses to test the study hypotheses and test for other relationships of interest. For the planned logistic regressions for which there were fewer than five events per condition, we described the results in lieu of reporting the inferential statistics due to potential for bias (27). The primary predictor variable was whether the patient received an IBH consultation the same day as a positive screen on the ASQ (same-day IBH encounter). The primary outcome variables were same-day and 31-day ED visits. Additional outcome variables included same-month IBH utilization and same-month PCP utilization. We included a number of potential covariates, including age, biological sex assigned at birth, race/ethnicity, PHQ-9 score, and insurance type. To select covariates for the final regressions, we conducted a series of univariate regressions with each potential covariate as the sole predictor (28, 29). Predictors with *p* < 0.2 were retained as covariates. When possible, chi-square analyses were conducted if the nature of the data precluded regression analysis.

## Results

### Sample characteristics

Table 2 reports the characteristics for three samples: (1) all patients who were screened, (2) patients who screened positive (non-acute or acute) on the ASQ, and (3) patients who screened acute positive on the ASQ. Age in years at the time of the ASQ, biological sex assigned at birth, and insurance plan were available for all participants. Race and ethnicity information that was reported as “unknown” or “declined” was categorized as missing (rates reported in Table 2 for each sample).

**Table 2:** Participant-level characteristics.

	**Total participant sample (*N* = 3,649)**	**Participants with at least one non-acute positive (*n* = 669)**	**Participants with at least one acute positive (*n* = 71)**
Age in years, *M* (*SD*)	13.99 (2.25)	14.98 (2.03)	14.69 (2.04)
Female sex assigned at birth, *n* (%)	2,151 (54%)	478 (71%)	59 (83%)
Race			
American Indian/Alaska Native, *n* (%)	64 (2%)	20 (3%)	2 (3%)
Asian, *n* (%)	569 (16%)	72 (11%)	5 (7%)
Black, *n* (%)	219 (6%)	40 (6%)	5 (7%)
White, *n* (%)	2,321 (64%)	443 (66%)	47 (66%)
Multiracial, *n* (%)	56 (2%)	11 (2%)	1 (1%)
Other Pacific Islander, *n* (%)	28 (1%)	6 (1%)	1 (1%)
Unknown/declined, *n* (%)	389 (11%)	21 (12%)	10 (14%)
Ethnicity, Hispanic, *n* (%)	899 (25%)	154 (25%)	23 (32%)
Race/Ethnicity is Hispanic and/or not White, *n* (%)	1,508 (41%)	241 (36%)	27 (38%)
Insurance, Public, *n* (%)	1,539 (42%)	304 (45%)	40 (56%)
PHQ-9, *M* (*SD*)	5.21 (5.79)	13.33 (6.48)	19.59 (6.27)

ASQ, Ask Suicide-Screening Questions; PHQ, Patient Heath Questionnaire.

**Figure 1:**
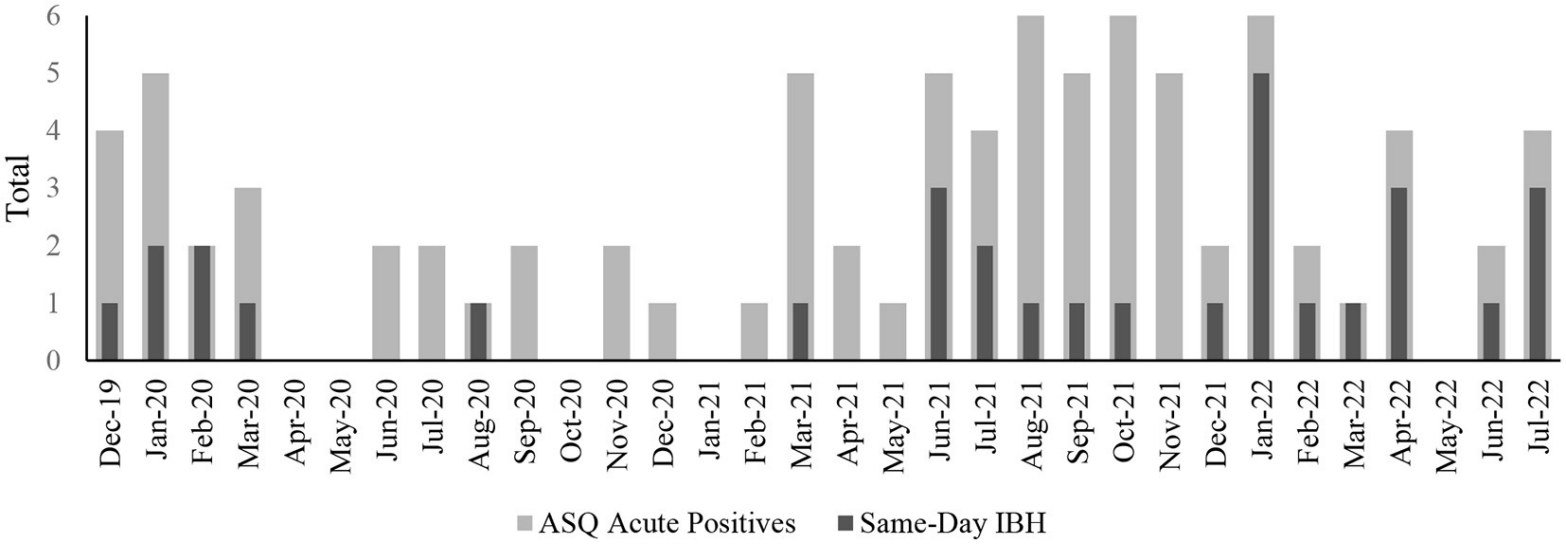
Number of positive acute suicide risk screenings and receipt of same-day integrated behavioral health (IBH) consultations during the study period.

Some patients in the sample completed the ASQ multiple times during the study period, yielding 7,982 unique ASQ scores from 3,649 unique patients. Out of all screenings, 1,380 (18%) were non-acute positive (ASQ questions 1–4 ≥ 1, ASQ question 5 = 0), from 669 unique patients (*mean* number of non-acute positive ASQs per patient = 2.20, *mode* = 1). A total of 87 ASQs (1%) acute positive (ASQ question 5 = 1) and these were from 71 unique patients (*mean* number of acute positive ASQs per patient = 1.23, *mode* = 1). Of the 1,469 combined positive ASQ scores, 165 (11%) received a same-day IBH consult. Among patients who screened acutely positive on the ASQ, 31 (36%) received a same-day IBH consultation. Figure 1 depicts the frequency of acute-positive ASQs and same-day IBH consultation by month over the course of the study period. In the positive/acute combined sample, higher (more severe) PHQ-9 score and older age both significantly predicted receipt of a same-day IBH consult (*p*<*0*.001 for both) and patients with public insurance were more likely to receive an IBH consultation, χ^2^ = (1, 1,469) = 11.99, *p* < 0.001. Neither binary racial and ethnic identity, χ^2^ = (1, 1,274) = 0.32, *p* = 0.57, or legal sex, χ^2^ = (1, 1,469) = 0.36, *p* = 0.54, were associated with receipt of a same-day IBH consultation.

### Emergency department utilization

During the study period, 129 patients had 163 documented ED visits (*mode* = 1, *range* = 1–12), 18 of which were related to suicidality. Table 3 displays the results of binomial logistic regressions with same-day IBH predicting same-day ED visits for participants with ASQ ≥ 1 (acute and non-acute positive screens together). Among all positive ASQ screens, same-day IBH was significantly associated with increased odds for all same-day ED visits and suicide-related same-day ED visits (*p* < 0.05). We were not able to run logistic regressions for the acute positive sample or with same-month ED visits as the outcome for the ASQ ≥ 1 sample due to insufficient events per condition. Table 4 outlines descriptive breakdowns of ED visits by ASQ result and receipt of IBH consultation. In all cases, a larger percentage of patients who had had same-day IBH contact had subsequent ED visits, but statistical significance could not be tested in most cases (although whether the differences are statistically significant is unknown in most cases). Among the 56 patients with an acute positive ASQ who did not receive an IBH consultation, one (2%) was admitted to the ED for suicidality in the following 31 days. Among the 31 who did receive a same-day IBH consultation, none were admitted to the ED for suicidality in the following 31 days.

**Table 3:** Regression of same-day emergency department admission on same-day integrated behavioral health encounters for patients screening positive for suicide risk.

	**All emergency department visits**			
		**95% CI**		
Variable	OR	LL	UL	*p*
Intercept	0.00	0.00	0.01	< .001
**Same-day IBH**	**10.64**	**2.95**	**42.62**	**< .001**
PHQ-9	1.09	.98	1.23	.117

*n* = 1,423 (n = 44 removed due to missing PHQ-9); OR, Odds Ratio; CI, Confidence Interval; LL, lower limit; UL, upper limit; ED, emergency department; IBH, integrated behavioral health. Statistically significant predictors are highlighted in bold.

**Table 4:** Emergency department visits by suiciding screening result and receipt of same-day integrated behavioral health consultation.

	**Total**	**Emergency department visits**			
		**Same day all**	**Same day suicide-related**	**Same month all**	**Same month suicide-related**
Acute positive ASQ, *n* (%)	87 (100%)				
Same-day IBH	31 (36%)	4 (12%)	4 (12%)	1 (3%)	0 (0%)
No Same-day IBH	56 (64%)	2 (3%)	1 (2%)	1 (2%)	1 (2%)
All positive ASQ, *n* (%)	1,469 (100%)				
Same-day IBH	165 (11%)	7 (4%)	5 (3%)	4 (2%)	1 (.06%)
No same-day	1,304 (89%)	7 (.05%)	2 (.02%)	22 (2%)	5 (.04%)

IBH, integrated behavioral health.

### Integrated behavioral health utilization

Among the 1,467 positive/acute ASQ screens, 327 (22%) received at least one additional contact with IBH within the next 31 days. Table 5 displays the results of a binomial regression with same-day IBH predicting 31-day IBH utilization for patients with positive ASQ screenings. Same-day IBH significantly and positively predicted subsequent 31-day IBH contacts, as did higher PHQ-9 score and race/ethnicity (racial and/or ethnic minority-identifying more likely to have a 31-day IBH visit).

**Table 5:** Regression of integrated behavioral health encounters within 31 days of positive suicide risk screening on integrated behavioral health encounters the same day as screening.

		**95% CI**		
**Variable**	**OR**	**LL**	**UL**	***p***
Intercept	0.25	0.06	0.95	.045
**Same-day IBH**	**11.70**	**7.71**	**18.04**	**< .001**
Age	0.92	0.85	1.01	.071
Sex	1.07	0.73	1.60	.729
**Race**	**1.54**	**1.13**	**2.11**	**.006**
Insurance	1.15	.84	1.58	.378
**PHQ-9**	**1.04**	**1.01**	**1.07**	**.002**

*n* = 1,423 (*n* = 44 removed due to missing PHQ-9); OR, Odds Ratio; CI, Confidence Interval; LL, lower limit; UL, upper limit; IBH, integrated behavioral health. Statistically significant predictors are highlighted in bold.

### Medical primary care utilization

Among patients who screened positive on the ASQ, 358 (24%) had a contact with a PCP within the next 31 days. Table 6 displays the results of a binomial regression with same-day IBH predicting 31-day PCP utilization for patients with positive ASQ screenings. Same-day IBH positively predicted at least one visit with the PCP in the following 31 days, as did race/ethnicity (racial and/or ethnic-minority identifying patients were more likely to have a 31-day PCP visit).

**Table 6:** Regression of medical primary care encounters within 31 days of positive suicide risk screening on integrated behavioral health encounters the same day as screening.

		**95% CI**		
**Variable**	**OR**	**LL**	**UL**	***p***
Intercept	0.80	0.25	2.55	.712
**Same-day IBH**	**2.84**	**1.92**	**4.20**	**< .001**
**Age**	**.91**	**0.84**	**.98**	**.010**
**Race/Ethnicity**	**1.46**	**1.11**	**1.92**	**.007**
Insurance	1.12	0.85	1.48	.430
PHQ-9	1.01	.99	1.04	.204

*n* = 1,469; OR, Odds Ratio; CI, Confidence Interval; IBH, integrated behavioral health. Statistically significant predictors are highlighted in bold.

### Mortality

There was a single recorded death in the sample due to non-suicide causes.

## Discussion

Integration of behavioral health services into pediatric primary care has grown substantially in recent years in recognition of its promise for meeting the increasing behavioral health needs of the population (30, 31). We sought to assess how IBH consultations in the context positive suicide screenings in pediatric primary care relate to subsequent use of services to address suicidality, including ED visits, follow-up IBH, and primary care. Nearly one-in-five patients screened non-acute or acute positive in our sample. Despite the high incidence of suicidality, less than 1% of screenings resulted in an ED visit and no patients died from suicide. These findings counter common concerns from providers and families that suicide screening may be iatrogenic or lead to marked increases in ED utilization (32–34). This study replicated a previous finding that over 90% of screen-positive youth who received a same-day IBH consult were diverted from the ED on both the day of their positive screen and during the subsequent month (7). However, we found that youth with positive screens who did not receive same-day IBH were also diverted from the ED at high rates. Indeed, contrary to our hypothesis, youth who received same-day IBH consultation after a positive suicide screen were more likely to be admitted to the ED that same day than their peers who did not receive an IBH consultation.

These results should be interpreted in the context of the broader roll-out of ASQ screening and systematic risk determination in the clinics under study. In contrast to Mancini and colleagues (7), who compared rates of IBH-associated ED diversion to a pre-IBH norm of referring all youth with positive screens to the ED, medical providers in our study did not refer all positive screens to the ED as a matter of course. Consistent with the U.S. National Strategy for Suicide Prevention (35), medical personnel in our clinic were trained in a secondary assessment process as part of implementing the ASQ. Beyond direct training, the presence of IBH in primary care is associated with PCP's increased confidence in managing mental health issues independently. Further, IBH-to-PCP “curbside” consultations may have occurred for some of the patients who did not receive face-to-face IBH consultations (36). We believe it is likely that medical providers prioritized patients they perceived as at higher-risk IBH consults, which is supported by the positive relationship between PHQ-9 scores and receipt of IBH consults (37).

The results suggest a unique value-add of IBH may be linking patients with suicide risk to short-term follow-up services. Patients in this study were more likely to engage with IBH in the month following their positive screen when they received a same-day IBH consultation. This finding is consistent with a recent study of same-day IBH responses to an array of behavioral health concerns in adolescent primary care, which found that patients who received a same-day consult were more likely to return for follow-up behavioral health in that clinic (38). Further, these results are consistent with the theoretical framework that IBH can address inequalities in care, reduce stigma, build rapport, and increase engagement (39–41). The relatively quick connection to follow-up in our study is consistent with 2012 Office of the Surgeon General's National Strategy for Suicide Prevention objective to provide timely access to care (35), particularly in the context of significant waitlists for longer-term care in the community at the time of the study, which has only increased in the post-pandemic surge in demand for behavioral health resources (42).

Patients who received a same-day IBH consultation were also more likely to contact a PCP in the subsequent month, even when controlling for depression severity. To our knowledge, the relationship between IBH and primary care utilization in the context of suicide risk has not previously been examined in pediatric populations, but other research has demonstrated better short-term attendance at medical appointments following same-day IBH consultations (i.e., “warm handoffs”) more generally (43). While longer-term reduction in utilization of unnecessary or inappropriate care would be a positive outcome of IBH, in the context of our at-risk sample, short-term increases in utilization may represent an appropriate level of care. Follow-up with a PCP in the month after a positive suicide screen allows for additional caring contacts (44), updates to safety plans, identification of changes in risk, and management of psychiatric medications or medical comorbidities, which are more likely among youth with suicidal ideation and attempts (45). Previous research suggests that PCPs' follow-up care for suicide risk is inconsistent and clinical guidelines are needed (45).

Importantly, we found that the relationship between same-day IBH and subsequent-month IBH and PCP follow-up was stronger for patients who self-identified as racial and/or ethnic minorities. While the reasons for this relationship are unclear, it aligns with the aim of IBH to increase accessibility and quality of care for historically underserved youth (40). We dichotomized race and ethnicity given the low incidence of our outcomes of interest. However, we acknowledge there is great variation in the social and material experiences of individuals from different identity groups that warrant exploration. For example, suicide rates among Black youth increased over the past 20 years faster than any other group, such that they are twice as likely to die by suicide (46, 47). Our results are consistent with other findings indicating that higher-integration IBH models may hold particular benefits for people from traditionally under-served groups (48). Future research should explore possible explanations of this dynamic, including familiarity with the primary care setting, reduction of stigma, provision of culturally responsive care, and logistical accessibility.

This study highlights some of the challenges of IBH implementation and areas for refinement of this model. Just over one third of acute-positive screens were followed by a same-day face-to-face IBH consult, considerably lower than expected even considering the apparent impact of COVID-19 social distancing restrictions. For comparison, Pereira and colleagues reported same-day consultations with 69% of acute-positive patients (14), whereas Mancini and colleagues reported 100% (7). We observed high variation in the proportion of acute-positive screens that received IBH consult across time, with the lowest proportions generally following the onset of the COVID-19 pandemic. Disruptions to medical systems during the peak of the pandemic may have impacted successful IBH integration at multiple levels (e.g., clinic distancing and space restrictions, reluctance to engage the ED) that took time to remediate. Established methods for increasing warm handoffs may be useful in such circumstances (49). Further study of the implementation process and quality improvement efforts to increase the rate of contact with IBH for youth with positive suicide screens is merited.

### Limitations and future directions

This study possesses notable limitations inherent to retrospective research. Group membership on the primary independent variable, receipt of same-day IBH consult, was non-random and subject to a number of potential confounds. Barriers to same-day IBH consultations were not formally assessed but may be attributable to limitations in IBH provider availability, high medical providers self-competence, or other unknown factors, such as existing mental health care. Further, it remains unclear whether and how the risk assessment and response process varied across IBH and medical providers. Future research should examine the specific content of suicide-risk responses to determine whether particular strategies impact outcomes. Data were limited to one healthcare system in large metro area under study. Future studies should compare clinics with IBH services to those without, to assess the indirect impact of IBH services on PCP behavior and subsequent ED admissions. Additionally, it is possible that patients presented to another ED or utilized other healthcare in the month following screening. When considering generalizability, it is important to note that the clinics under study are part of an academic medical center in a metropolitan area. Future work should explore how IBH may affect suicide screening and follow-up in clinics in non-academic settings and rural areas.

## Data availability statement

The original contributions presented in the study are included in the article/supplementary material, further inquiries can be directed to the corresponding author.

## Ethics statement

The study involving humans was approved by the Institutional Review Board at Oregon Health and Science University. The study was conducted in accordance with the local legislation and institutional requirements. The Ethics Committee/institutional review board waived the requirement of written informed consent for participation from the participants or the participants' legal guardians/next of kin because the study did not involve active participation, data collection was fully based on retrospective chart review.

## Author contributions

AR, BW, and EB contributed to the conception and design of the work and acquisition of the data. AR, BW, NW, EB, and MB contributed to the analysis and interpretation of the work, drafting the work, and critical revision, authors have provided approval for publication of the content and agree to be accountable for all aspects of the work in ensuring that questions related to the accuracy or integrity of any part of the work are appropriately investigated and resolved. All authors contributed to the article and approved the submitted version.

## References

[1] Centers for Disease Control & Prevention. Youth Risk Behavior Survey Data Summary & Trends Report, 2011–2021. Atlanta, GA (2023).

[2] Horowitz LM Ballard ED Pao M. Suicide screening in schools, primary care and emergency departments. Curr Opin Pediatr. (2009) 21:620–7. 10.1097/MOP.0b013e3283307a89 19617829 PMC2879582

[3] Milliman CC Dwyer PA Vessey JA. pediatric suicide screening: a review of the evidence. J Pediatr Nurs. (2021) 59:1–9. 10.1016/j.pedn.2020.12.011 33387798

[4] Helms SW Prinstein M. Risk assessment and decision making regarding imminent suicidality in pediatric settings. Clinic Pract Pediatric Psychol. (2014) 2:176. 10.1037/cpp0000048

[5] Horowitz LM Bridge JA Pao M Boudreaux ED. Screening youth for suicide risk in medical settings: time to ask questions. Am J Prev Med. (2014) 47:S170–5. 10.1016/j.amepre.2014.06.002 25145735 PMC8547061

[6] Diamond GS O'Malley A Wintersteen MB Peters S Yunghans S Biddle V. Attitudes, practices, and barriers to adolescent suicide and mental health screening: a survey of pennsylvania primary care providers. J Prim Care Community Health. (2012) 3:29–35. 10.1177/2150131911417878 23804852

[7] Mancini K Myers BR Pajek J Ramirez L Stancin T. Addressing suicide risk in primary care: cost savings associated with diverting patients from emergency departments. J Dev Behav Pediatr. (2023) 44:e19–e23. 10.1097/DBP.0000000000001141 36563342

[8] Jewell M Shehab S Kaplan R Fanton J Hettler J. Costs without value when treating pediatric behavioral patients in the Ed. NEJM Catalyst. (2022) 3(2). 10.1056/CAT.21.0332

[9] Dolan MA Fein JA Committee on Pediatric Emergency M. Pediatric and adolescent mental health emergencies in the emergency medical services system. Pediatrics. (2011) 127:e1356–66. 10.1542/peds.2011-0522 21518712

[10] Asarnow JR Babeva K Horstmann E. The emergency department: challenges and opportunities for suicide prevention. Child Adolesc Psychiatr Clin N Am. (2017) 26:771–83. 10.1016/j.chc.2017.05.002 28916013 PMC6768433

[11] Zeller SL Calma NM Stone A. Effect of a regional dedicated psychiatric emergency service on boarding and hospitalization of psychiatric patients in area emergency departments. Western J Emerg Med Integrat Emerg Care Populat Health. (2014) 15:17828. 10.5811/westjem.2013.6.17848 24578760 PMC3935777

[12] Case SD Case BG Olfson M Linakis JG Laska EM. Length of stay of pediatric mental health emergency department visits in the United States. J Am Acad Child Adolesc Psychiatry. (2011) 50:1110–9. 10.1016/j.jaac.2011.08.011 22023999 PMC3241993

[13] Hughes JL Anderson NL Wiblin JL Asarnow JR. Predictors and outcomes of psychiatric hospitalization in youth presenting to the emergency department with suicidality. Suicide Life Threat Behav. (2017) 47:193–204. 10.1111/sltb.12271 27371938

[14] Pereira LM Wallace J Brown W Stancin T. Utilization and emergency department diversion as a result of pediatric psychology trainees integrated in pediatric primary and specialty clinics. Clinic Pract Pediatric Psychol. (2020) 20:315. 10.1037/cpp0000315

[15] Horowitz LM Mournet AM Lanzillo E He J-P Powell DS Ross AM. Screening pediatric medical patients for suicide risk: is depression screening enough? J Adolesc Health. (2021) 68:1183–8. 10.1016/j.jadohealth.2021.01.028 33712380 PMC8154669

[16] Jimenez G Matchar D Koh GCH Tyagi S Van Der Kleij RMJJ Chavannes NH. Revisiting the four core functions (4cs) of primary care: operational definitions and complexities. Primary Health Care Research & Develop. (2021) 21:22. 10.1017/S1463423621000669 34753531 PMC8581591

[17] National Center for Injury Prevention and Control Centers for Disease Control and Prevention. Suicide Prevention Resource for Action: A Compilation of the Best Available Evidence. Atlanta, GA (2022).

[18] Heath B Wise Romero P Reynolds K. A Review and Proposed Standard Framework for Levels of Integrated Healthcare. Washington, DC: SAMHSA-HRSA Center for Integrated Health Solutions (2013).

[19] Brodsky BS Spruch-Feiner A Stanley B. The zero suicide model: applying evidence-based suicide prevention practices to clinical care. Front Psychiatr. (2018) 9:33. 10.3389/fpsyt.2018.00033 29527178 PMC5829088

[20] National Institute of Mental Health. Ask Suicide-Screening Questions (Asq) Toolkit. Available online at: www.nimh.nih.gov/ASQ.

[21] Horowitz LM Bridge JA Teach SJ Ballard E Klima J Rosenstein DL. Ask suicide-screening questions (Asq): a brief instrument for the pediatric emergency department. Arch Pediatr Adolesc Med. (2012) 166:1170–6. 10.1001/archpediatrics.2012.1276 23027429 PMC6889955

[22] Aguinaldo LD Sullivant S Lanzillo EC Ross A He JP Bradley-Ewing A. Validation of the ask suicide-screening questions (ASQ) with youth in outpatient specialty and primary care clinics. Gen Hosp Psychiatry. (2021) 68:52–8. 10.1016/j.genhosppsych.2020.11.006 33310014 PMC7855604

[23] Anand P Bhurji N Williams N Desai N. Comparison of Phq-9 and Phq-2 as screening tools for depression and school related stress in inner city adolescents. J Primary Care & Community Health. (2021) 12:21501327211053750. 10.1177/21501327211053750 34905994 PMC8679043

[24] Allgaier AK Pietsch K Fruhe B Sigl-Glockner J Schulte-Korne G. Screening for depression in adolescents: validity of the patient health questionnaire in pediatric care. Depress Anxiety. (2012) 29:906–13. 10.1002/da.21971 22753313

[25] Johnson JG Harris ES Spitzer RL Williams JB. The patient health questionnaire for adolescents: validation of an instrument for the assessment of mental disorders among adolescent primary care patients. J Adolesc Health. (2002) 30:196–204. 10.1016/S1054-139X(01)00333-0 11869927

[26] Kenny J Costello L Kelsay K Bunik M Xiong S Chiaravalloti L. All hands on deck: addressing adolescent depression in pediatric primary care. J Pediatric Psychol. (2021) 46:903–11. 10.1093/jpepsy/jsab033 34010421

[27] Vittinghoff E McCulloch CE. relaxing the rule of ten events per variable in logistic and cox regression. Am J Epidemiol. (2007) 165:710–8. 10.1093/aje/kwk052 17182981

[28] Bursac Z Gauss CH Williams DK Hosmer DW. Purposeful selection of variables in logistic regression. Source Code Biol Med. (2008) 3:17. 10.1186/1751-0473-3-17 19087314 PMC2633005

[29] Ranganathan P Pramesh C Aggarwal R. Common pitfalls in statistical analysis: logistic regression. Perspect Clinic Res. (2017) 8:148. 10.4103/picr.PICR_123_17 28828311 PMC5543767

[30] Foy JM Green CM Earls MF. Mental health competencies for pediatric practice. Pediatrics. (2019) 144:e20192757. 10.1542/9781610023658-part08-mental_health 31636143

[31] Richman EL Lombardi BM Zerden LD. Mapping colocation: using national provider identified data to assess primary care and behavioral health colocation. Famil Syst Health. (2020) 38:16. 10.1037/fsh0000465 32202831

[32] Ballard ED Stanley IH Horowitz LM Cannon EA Pao M Bridge JA. Asking youth questions about suicide risk in the pediatric emergency department: results from a qualitative analysis of patient opinions. Clinic Pediatric Emerg Med. (2013) 14:20–7. 10.1016/j.cpem.2013.01.001 23908599 PMC3725561

[33] Horowitz L Ballard E Teach SJ Bosk A Rosenstein DL Joshi P. Feasibility of screening patients with nonpsychiatric complaints for suicide risk in a pediatric emergency department: a good time to talk? Pediatric Emerg Care. (2010) 26:787. 10.1097/PEC.0b013e3181fa8568 20944511 PMC3298546

[34] Grupp-Phelan J McGuire L Husky MM Olfson M. A randomized controlled trial to engage in care of adolescent emergency department patients with mental health problems that increase suicide risk. Pediatric Emerg Care. (2012) 28:1263–8. 10.1097/PEC.0b013e3182767ac8 23187979

[35] General Office of the Surgeon General and the National Office for Suicide Prevention. National Strategy for Suicide Prevention: Goals and Objectives for Action: A Report of the US Surgeon General and of the National Action Alliance for Suicide Prevention (2012).

[36] Olufs EL Valleley RJ Hembree KC Evans JH. Brief educational “curbside consultation”: impact on attention-deficit/hyperactivity disorder referrals in an integrated healthcare setting. Famil Syst Health. (2016) 34:221. 10.1037/fsh0000210 27632542

[37] Aarons GA Sommerfeld DH Hecht DB Silovsky JF Chaffin MJ. The impact of evidence-based practice implementation and fidelity monitoring on staff turnover: evidence for a protective effect. J Consult Clin Psychol. (2009) 77:270–80. 10.1037/a0013223 19309186 PMC2742697

[38] Anand P Desai N. Correlation of warm handoffs versus electronic referrals and engagement with mental health services co-located in a pediatric primary care clinic. J Adolesc Health. (2023) 10.1016/j.jadohealth.2023.02.032 37061906

[39] Shahidullah JD Hostutler CA Coker TR Allmon Dixson A Okoroji C Mautone JA. Child health equity and primary care. Am Psychol. (2023) 78:93. 10.1037/amp0001064 37011162

[40] Arrojo MJ Bromberg J Walter HJ Vernacchio L. Pediatric primary-care integrated behavioral health: a framework for reducing inequities in behavioral health care and outcomes for children. Pediatric Clinics. (2023) 10.1016/j.pcl.2023.04.004 37422314

[41] O'Loughlin K Donovan EK Radcliff Z Ryan M Rybarczyk B. Using integrated behavioral healthcare to address behavioral health disparities in underserved populations. Translat Issues Psychologic Sci. (2019) 5:374. 10.1037/tps0000213

[42] Sowa NA Zeng X. A comprehensive examination of pediatric behavioral health service demand and utilization in a large, academic health system from 2019 to 2021. Psychiatric Quarter. (2023) 1–9. 10.1007/s11126-023-10030-1 37219750 PMC10203664

[43] Young ND Mathews BL Pan AY Herndon JL Bleck AA Takala CR. Warm Handoff, or Cold Shoulder? an analysis of handoffs for primary care behavioral health consultation on patient engagement and systems utilization. Clinic Pract Pediatric Psychol. (2020) 8:241–6. 10.1037/cpp0000360

[44] Skopp NA Smolenski DJ Bush NE Beech EH Workman DE Edwards-Stewart A. Caring contacts for suicide prevention: a systematic review and meta-analysis. Psychologic Serv. (2023) 20:74. 10.1037/ser0000645 35420858

[45] Butwicka A Frisén L Almqvist C Zethelius B Lichtenstein P. Risks of psychiatric disorders and suicide attempts in children and adolescents with type 1 diabetes: a population-based cohort study. Diabetes care. (2015) 38:453–9. 10.2337/dc14-0262 25650362 PMC4338504

[46] Bridge JA Horowitz LM Fontanella CA Sheftall AH Greenhouse J Kelleher KJ. Age-related racial disparity in suicide rates among Us Youths from 2001 through 2015. JAMA pediatrics. (2018) 172:697–9. 10.1001/jamapediatrics.2018.0399 29799931 PMC6137506

[47] Sheftall AH Vakil F Ruch DA Boyd RC Lindsey MA Bridge JA. Black youth suicide: investigation of current trends and precipitating circumstances. J Am Acad Child Adolesc Psychiatr. (2022) 61:662–75. 10.1016/j.jaac.2021.08.021 34509592 PMC8904650

[48] Chakawa A Belzer LT Perez-Crawford T Brei N. Which model fits? evaluating models of integrated behavioral health care in addressing unmet behavioral health needs among underserved sociodemographic groups. Evid Based Pract Child Adolesc Mental Health. (2020) 5:251–70. 10.1080/23794925.2020.1796549

[49] Germán M Hsu-Walklet T Gurney BA Parekh J Stein Berman R Herrick J. “Nice to meet you”: a quality improvement project to increase warm handoffs. Clinic Pract Pediatr Psychol. (2020) 8:247–56. 10.1037/cpp0000357

